# Non-tuberculous mycobacteria isolated from slaughter pigs in Mubende district, Uganda

**DOI:** 10.1186/1746-6148-8-52

**Published:** 2012-05-07

**Authors:** Adrian Muwonge, Clovice Kankya, Tone B Johansen, Berit Djønne, Jacques Godfroid, Demelash Biffa, Vigdis Edvardsen, Eystein Skjerve

**Affiliations:** 1 Centre for Epidemiology and Biostatistics, Norwegian School of Veterinary Science, P.O. Box 8146 Dep, 0033, Oslo, Norway; 2 Department of Biosecurity, Ecosystems and Public Health (BEP), College Of Veterinary Medicine, Animal Resources & Biosecurity Makerere University, P.O. Box 7062, Kampala, Uganda; 3 Norwegian Veterinary Institute, P.O. Box 750, N-0106, Oslo, Norway; 4 Section for Arctic Veterinary Medicine, Norwegian School of Veterinary Science, Stakkevollveien 9010, Tromsø, Norway; 5 College of Medicine, University of Arizona, 1656 E. Mabel St, P.O. Box 245221, Tucson, AZ, 85724, USA

## Abstract

**Background:**

The importance of infections caused by non-tuberculous mycobacteria (NTM) in animals and humans has gained considerable recognition during the past few years. In the developed world, where pig production is extensively practiced, studies on mycobacterial infections and related control strategies have received increasing attention. The infections are reported to be caused by a wide spectrum of NTM. Unfortunately, these infections have been less recognized in sub-Saharan Africa owing to lack of awareness and systematic studies. In this study we aimed at isolating and identifying species of mycobacteria involved in causing infections in slaughter pigs in Mubende district of Uganda. Furthermore we wanted to identify factors associated with infection prevalence in the study area.

**Methods:**

A total of 363 lymph nodes were collected and cultured for the presence of mycobacteria. Isolates were identified by 16S rDNA gene sequencing. A questionnaire survey was administered to identify production related factors associated with infection prevalence. Data were assembled and analysed using descriptive statistics and mixed effects logistic regression analysis.

**Results:**

Mycobacteria were detected in 39 % (143/363) of the examined lymph nodes, 63 % (59/93) of lymph nodes with gross lesions typical of mycobacteriosis and 31% (84/270) of lymph nodes with no visible lesions. Nineteen per cent of the isolated mycobacteria were identified as *Mycobacterium (M) avium*, of these 78% and 22% were *M. avium* sub sp. *Hominissuis* and *avium* respectively. Other mycobacterial species included *M. senuense* (16%)*, M. terrae* (7%) and *M. asiaticum* (6%). This study found free range systems (OR = 3.0; P = 0.034) and use of water from valley dams (OR = 2.0; P = 0.049) as factors associated with high prevalence of mycobacteria in slaughter pigs.

**Conclusions:**

This study demonstrated a high prevalence of NTM infections among slaughter pigs in Mubende district of Uganda. *M. avium* was the most prevalent of all NTM isolated and identified. Free range system of pig management and valley dam water were the most significant factors associated with NTM prevalence in Mubende district. These findings could be of a major public health concern given that it is in a predominantly pork consuming population with 18% HIV/AIDS prevalence. Therefore, stringent post-mortem inspection at the slaughter houses is of paramount importance to reduce human exposure.

## Background

Non-tuberculous mycobacteria (NTM) are the most prevalent type of mycobacteria. Unlike members of the *Mycobacterium tuberculosis* complex (MTC) that are highly pathogenic, the majority of NTM have been regarded as non-pathogenic. This opinion is now changing as more NTM have been associated with several human diseases [1]. NTM naturally occur in the environment, especially water and soil, thus considered to be ubiquitous microorganisms [2]. The absence of documented host-to-host transmission has led to the belief that environment is the primary source of NTM infections for animals as well as humans. Currently, it is estimated that 50% of the AIDS patients are likely to develop infections due to *Mycobacterium Avium* Complex (MAC) predominately *M. avium,* if and when severely immunocompromised [3]. Other NTM reported to cause disease include; *M. simiae* which is associated with lung disease in immune compromised patients and *M. terrae* isolated from patients with urinary tract infections and chronic tenosynovitis [4]. Reports documenting prevalence are predominantly from developed countries [5-7]. In the Netherlands and Croatia for example, *M. avium* and *M. avium* subsp. *hominissuis* accounted for up to 39.8% and 93% of mycobacteria isolated from porcine lymph nodes respectively [8,9]. There are few studies documenting the prevalence of NTM in developing countries especially in sub-Saharan Africa, however, available reports indicate that the problem could be more comprehensive than previously documented [10-12].

Pig mycobacterial infections are characterized by chronic inflammatory reactions in various body parts, but mostly in the digestive system. Calcified tubercles, inflamed lymph nodes and sarcoid-like granulomas are the most common feature of this disease [5,10,13]. The infection usually has a benign course and diagnosis is usually only possible during post-mortem examinations at slaughter [6,10]. Studies in Switzerland and Nigeria showed that NTM can be isolated from lymph nodes which appear healthy [13,14]. Although these reports seem to suggest that a considerable amount of mycobacteria could be missed at inspection, stringent meat inspection practice is still the key to reducing the human exposure. Given the lack of appropriate meat inspection practices in majority of sub-Saharan African countries, human exposure to NTM through meat seems to be a viable route. Studies have shown a genetic relatedness between *M. avium* isolates from humans and pigs, adding to speculations that pigs may be a vehicle for NTM infection in humans [9,13,15,16]. Production systems are known to play a key role in the distribution of NTM [13]. In Nigeria, it is reported that pigs are mainly infected by ingestion of soil, litter, dust contaminated by faeces of tuberculous chicken or consumption of improperly processed infected chicken [13]. On the other hand reports from Czech Republic and Lithuania indicated that outbreaks in herds have been due to contaminated peat, compost and saw dust [17,18]. Therefore, identification of mycobacterial species involved in pig mycobacteriosis is important for prevention and control strategies.

Molecular sequencing using 16S rDNA has particularly been used in identification of bacteria with unusual phenotypic profiles and slow growing rates. Not only has it provided insight in aetiologies of diseases but also led to identification of novel mycobacterial species [19]. In Uganda, studies have shown a steady increase in pork consumption, especially in urban areas. Most of the pigs consumed trace their origin back to rural areas [20,21] such as Mubende. Pigs are mostly kept on free range and considered low-input livestock which grow to market size on minimal feed inputs [21]. In Mubende, a high prevalence of HIV/AIDS (18%) combined with malnutrition has resulted in a high proportion of immune compromised individuals in the population [20]. Recent reports also show an increase in NTM infections in HIV/AIDS patients [22,23], and the large population of free ranging pigs in a predominantly pork eating population has added to speculations that pigs could play a role in the dissemination of NTM in human populations [13,15]. There is a need to identify the types of mycobacteria prevalent among the slaughtered pig. Therefore, the aim of this study was to isolate and identify mycobacterial species prevalent among slaughtered pigs in Mubende district. Furthermore, to identify production related factors associated with high prevalence of NTM in the pig population in the area.

## Results

Out of 363 collected and cultured lymph nodes, 93 (26%) had grossly visible pathological lesion suggestive of mycobacteriosis, while 270 (74%) showed no visible lesions. Important pathologic characteristics of the lesions included caseous and non caseous granulomatous lesions, inflamed lymph nodes, a sarcoid-like granuloma and cyst like lesions.

Mycobacteria were detected in 143 (39.3 %) of the samples, both in lymph nodes showing macroscopic lesions (63.4%) and in lymph nodes without visible lesions (31.4%). The proportion of samples containing mycobacteria varied significantly between the studied sub-counties, a high (58%) and low (20%) prevalence were recorded in Kiyuni and Kiganda respectively (Table 1). There was no salient difference in prevalence of NTM between pigs slaughtered in open and closed slaughtered houses (Table 1).

**Table 1:** Non-tuberculous mycobacteria isolated from the sub-maxillary lymph nodes of pigs at slaughter in Mubende district

**Origin of sample**	**No of samples**	**No / % of samples containing mycobacteria**
Lymph nodes with lesion	93	59 / 63.4%
Lymph nodes without lesions	270	84 / 31%
Open slaughterhouse	223	87 / 39.0%
Closed slaughterhouse	140	56 / 40%
Madudu	113	62 / 54.8%
Kiyuni	35	20 / 58.8%
Kiganda	10	2 / 20.0%
Town council	69	19 / 27.5%
Kassanda	11	5 / 45.5%
Kasambya	16	4 / 25.0%
Bagezza	18	6 / 33.3%
Myanzi	20	4 / 20.0%
Butologo	28	11 / 39.3%
Bukuya	43	10 / 23.3%
**Total**	363	143 / 38.8%

**Figure 1:**
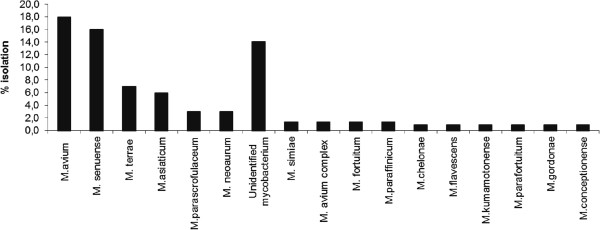
**Relative frequency of *Mycobacterium *spp. isolated from slaughtered pigs in Mubende district, Uganda.**

*M. avium* was the most predominant mycobacterial species identified, accounting for 19% (27/143) of the isolates. Further analysis revealed that 78 % (21/27) of *M. avium* were *M. avium* subsp. *hominissuis* and 22% (6/27) were *M. avium* subsp. *Avium* (Table 2). *M. senuense, M. terrae* and *M. asiaticum* accounted for 16%, 7%, and 6% of the total number of mycobacterial isolates, respectively (Figure 1). In addition, other species including *M. simiae, M. chelonae* and *M. gordonae* were found. Fourteen per cent (20/143) could not be identified because they did not have a match with any reference strain identification in the National Centre for Biotechnology Information (NCBI) database. Associations between variables recorded in the questionnaire and prevalence of NTM in the pigs are shown in Table 3. More mycobacteria were isolated from lymph node samples with grossly visible macroscopic lesions compared to those with no visible lesions (OR = 3.0; P < 0.001). Pigs whose source of drinking water was valley dams or water holes harboured more mycobacteria than those getting water from boreholes (OR = 3.0, P = 0.014; OR = 2,0, P = 0.049, respectively). There was a significantly higher prevalence of free rang pigs harbouring mycobacteria compared to pigs housed in-doors (OR = 3.0; P = 0.034). On the other hand, pigs that were reared in predominantly rocky/sandy areas were more likely to harbour mycobacteria compared to pigs reared in loam and clay soil areas. The mixed-effect logistic model also showed a significant variation in mycobacteria isolated between the studied sub-counties than within sub-counties (Table 3).

**Table 2:** **Shows *M.avium *characterisation to Sub-species’ level using IS 1245 and IS 901 PCR**

**Label**	***M.avium *ACCU-PROBE**	**PCRIS 1245**	**PCRIS 901**	**ID**	**%**
+	27	27	6	Maa	22
-	116	0	21	Mah	78
Total	143	27	27	-	19

Maa is *Mycobacterium avium* sub species *avium* and Mah is *Mycobacterium avium* sub *species hominissuis.*

**Table 3:** Mixed effects logistic regression model for factors associated with mycobacteria detected in pigs at slaughter in Mubende District of Uganda

**Variables**	**Level**	**Proportion %**	**Odds ratio**	**P-value**	**95 % CI**	**Variance estimate**
Lymph node pathology	No lesions	40	1			
	Lesion	60	3.0	0.001	(1.55-5.48)	
Type of water source	Bore holes	17.6	1	-	-	
	Valley dams	31.3	2.0	0.049	(1.09-5.15)	
	Water holes	51.1	3.0	0.014	(1.32-7.31)	
Pig management system	Housed	17.7	1	-	-	
	Free range	46.8	3.0	0.034	(1.22-5.97)	
Soil type in area of isolation	Rocky	8.5	1	-	-	
	Loam	46.1	0.12	0.001	(0.04-0.39)	
	Clay	44.6	0.05	0.009	(0.005-0.52)	
Month of sampling	September	18.4	1	-	-	
	October	16.3	0.11	0.001	(0.03-0.33)	
	November	14.1	0.13	0.001	(0.04-0.42)	
	January	14.2	0.3	0.008	(0.09-0.93)	
Random effect	Sub county		-	-	0.19-2.59)	0.86

## Discussion

This study found that mycobacteria were present in 39% of cultured lymph nodes from slaughtered pigs in Mubende district of Uganda. Sixty three per cent were from lymph nodes with macroscopic lesions compatible with mycobacteriosis and 31.4% were from those without lesions. This was higher than the 21% and 14% isolated from lymph nodes of slaughtered pigs in Nigeria and Switzerland, respectively [13,14]. This difference could be attributed to pig management practices. Pigs in industrialised countries like Switzerland and Lithuania are mainly kept indoors, and the exposure rate will therefore probably be lower and in cases where it occurs it is reported to be due to mycobacteria in water, peat and animal bedding [18]. Unlike the pigs in Nigeria, majority of pigs in Mubende district were reared using free or semi free range management systems. The role of management system in NTM prevalence was further consolidated by the evidence from the regression analysis of data which showed a significantly higher level of mycobacteria in free range or tethered pigs compared to housed pigs (OR = 3; P = 0.034). Free ranging pigs have a higher risk of exposure to environmental mycobacteria owing to their food scavenging behaviour. They are not provided with supplementary feeds and as a result are often malnourished and infected by different microorganisms, parasites, and possibly predisposing them to mycobacterial infections. This is in accordance with findings from other studies [1,23,24].

This study revealed that pigs from homesteads with valley dams or water holes as sources of water harboured significantly more mycobacteria than those with boreholes as water sources (OR = 2.0; 3.0 and P = 0.049; 0.014) as shown in Table 3. In a previous study carried out in the same environments, more mycobacteria from these open water sources were detected than from boreholes [23]. Given that valley dam water is harvested as storm water from the hilly terrains, it is therefore expected to be highly loaded with microbes including mycobacteria [1,2]. Such water is often shared by humans, livestock and wildlife, and thus constitutes a primary source of numerous infections including NTM.

Previous studies [5,10,13,14] have shown that *M. avium* is the most commonly detected *Mycobacterium* in lymph nodes of pigs. This is in agreement with the findings of the present study, where *M. avium* accounted for 19% of the isolated species. It is however lower than the 39.8% *M. avium* isolated from porcine lymph nodes in the Netherlands [8]. *M. avium* subsp. *hominisuis* were more prevalent than *M. avium* subsp. *avium* in this study, which is in agreement with previous studies [9]. In this study, *M. avium* subsp. *avium* was isolated from 4.3% (4/93) of the lymph nodes with lesions, and 2.1% from lymph nodes without macroscopic lesions, consolidating the findings from recent studies [18]. Although this is the first documentation of *M. avium* subsp. *avium* and *M. avium* subsp. *hominisuis* in pigs from Uganda, the former is known to be pathogen for birds and its presence in pigs is reported to indicate contact with infected birds [25]. This indeed could be the case given the free range and tethering system used for rearing pigs in Mubende district. Stringent post mortem inspection remains a critical control method in reducing the exposure risk to human populations. Given the HIV/AIDS status of Uganda, and the fact that 50% of the AIDS patients are likely to develop infections due to *M. avium* if their immune status is low [3], this finding is of public health interest.

Many different NTM other than *M. avium* were isolated from the pigs in the present study, most of these species have previously been also isolated from humans and pigs elsewhere [23,26]. For instance *M. simiae* has previously been reported to cause pulmonary infection in HIV patients [4,26,27]. In Uganda, *M. simiae* has recently been isolated from the environments inhabited by primates in Mubende district [23], and from febrile patients in Mulago referral hospital in Kampala [27]. *M. chelonae* is known to cause cutaneous infections especially in patients with AIDS [28,29] and *M. terrae* has recently been isolated from the environments in Mubende district [23], suggesting that these free range pigs acquire the infection from the environment. *M. terrae* has been isolated from patients with urinary tract infections and chronic tenosynovitis [4]. *M. senuense* has also been isolated from Mubende district before, although its role in disease causation is not well investigated. *M. asiaticum* was detected from pigs in the present study, but not from the environment in the same study area [23]. Its clinical significance has been well documented in Queensland Australia [30], where it was isolated from patients with extra pulmonary infections. In Queensland, the environment was reported to be the most probable source. *M. gordonae,* previously isolated from Mubende environments [23], is known to cause infections especially in patients with an underlying predisposition or immunosuppression such as AIDS, steroids therapy or patients undergoing peritoneal dialysis [4]. Petroleum by-product oxidising mycobacteria, *M. paraffinicum*[31] was also isolated from pigs.

This study found a higher prevalence of NTM in pigs from Madudu and Kiyuni and lower prevalence in Myanzi, Kiganda and Bukuya. This is could be due to the fact that Madudu and Kiyuni inherently have a high pig population thus apparently increasing the number of pigs exposed to the infection giving higher apparent prevalence of NTM. This is qualified by the large variation (VE < =0.86) between than with sub counties as shown by the regression model (Table 3).

In rural settings of Uganda, illegal slaughtering of pigs in the backyard and in unhygienic slaughterhouses is a common practice. The lack of strict regulations and enforcement mechanisms in meat inspection practice means that carcasses are usually washed with unhygienic water before being made ready for sale. This water is known to be heavily contaminated, particularly following the rainy season [23]. Therefore, it is possible that some of the mycobacteria isolated might be due to contamination rather than infection. However, their presence on the carcass may pose a health risk for meat consumers.

## Conclusions

This study documented a wide variety of NTMs species occurring at a prevalence as high as (39%) of cultured lymph nodes from slaughtered pigs in Mubende district, with 63% and 31% isolated from lymph nodes with and without gross lesions typical of mycobacteriosis respectively. Given that majority of these were *M. avium*, this could be of public health importance in this predominantly pork consuming population with 18% HIV/AIDS prevalence. Therefore, implementation of stringent post mortem inspection at the slaughter houses is of paramount importance in order to safeguard the public health.

## Methods

### Study district

**Figure 2:**
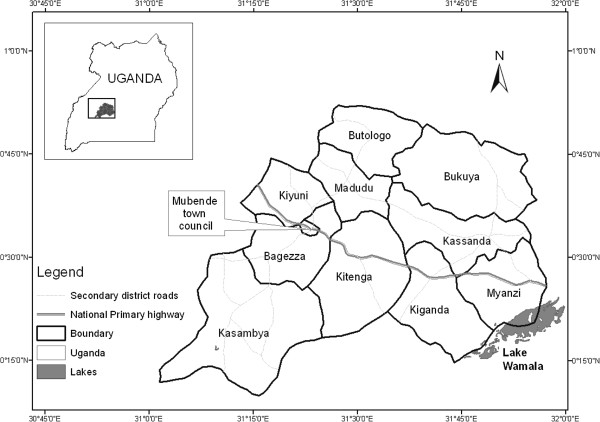
Map of Mubende district in Uganda, with the 10 sub-counties.

Mubende is located in the central region of the Ugandan cattle corridor and is divided into two counties; Buwekula and Kassanda. The counties are further sub-divided into 10 sub-counties; Bagezza, Butologo, Kasambya, Kitenga, Kiyuni, Madudu, Bukuya, Kassanda, Kiganda and Myanzi (Figure 2). The district lies at an altitude between 1372-1448 m above sea level, and pastoral and mixed agro-livestock ecosystems are the predominant modes of livestock production in the different altitude ranges [32]. Mubende is inhabited by approximately 750,000 people, most of whom live in urban and peri-urban areas [32,33]. Sixty-four per cent of the population lives below the poverty line. Pork industry is the most profitable venture in this area, and the district is one of the key sources of pork supply for the growing markets in Kampala, the capital city of Uganda [21,32].

### Study design and sampling strategy

The study design, sampling and sample size determination is described in an earlier study [12], However, in addition to the 93 cultured sub-maxillary lymph nodes with lesions compatible with mycobacterioses described in the previous study [12], 270 sub-maxillary lymph nodes without visible macroscopic lesions were cultured for detection of mycobacteria. The lymph nodes were collected in sterile, labelled sample bottles and transported in an icebox to laboratory at a minimum temperature of 4°C for culture of mycobacteria. In addition to lymph node collection, the pigs were traced back to their sources/farms and with consent a questionnaire was administered to the farmers.

### Ethical clearance

Scientific and ethical clearance for this study was obtained from the Uganda National Council for Science and Technology (UNCST). The research ethics committee found this study to be scientifically and ethically in accordance with the requirements and therefore was approved with reference number: HS 879.

### Isolation and identification of mycobacteria

Samples from the lymph nodes were collected and decontaminated, and mycobacteria were isolated as described earlier [12]. Acid fast bacteria, as demonstrated by Ziehl-Neelsen (ZN) staining, were identified as *Mycobacterium* spp, and further identified. Two loop-full of pure colonies from the second or third subculture were killed by heat treatment at 96°C for 20 minutes. Genomic DNA was isolated and characterized based on sequencing of the 16S rRNA gene using the following primers: 16S8F (AGAGTTTGATCMTGGYTCAG) and 16SM259 (TTTCACGAACAACGCGACAA) [23]. Isolates found to be *M. avium* were further confirmed by ACCU Probe (GenProbe Inc., San Diego, CA) and IS *1245* PCR. IS*901* PCR was used to differentiate between subspecies, as *M. avium* subsp. *hominissuis* (IS*901* negative) and *M. avium* subsp. *avium* (IS*901* positive) [34,35].

The obtained sequences were edited and analysed in the bioinformatics software Bio-edit ( http://www.mbio.ncsu.edu/BioEdit/bioedit.html) and the sequences were blasted at the NCBI Blast database (National Centre for Biotechnology Information). The species identification was strictly determined based on the maximum score and maximum identity values of the reference sequences in NCBI Blast alignment. Isolates with the maximum scores and maximum identities of 100 or 99% and documented as approved nomenclature were accepted for species identification [23].

### Data collection and analysis

Information obtained from the questionnaires and the corresponding laboratory results for individual pigs were assembled and validated using Excel® 2007. The data were statistically analysed using Stata (Stata/SE 11 for windows, Stata Corp, College Station, TX). Descriptive and summary analysis was used to estimate the prevalence of NTM infection with respect to presence or absence of acid fast bacteria. A univariable association between presence/absence of mycobacteria in the samples and the recorded individual variables was computed, and only variables that had (*p* value ≤ 0.25) from the univariable analysis were included in the final model. A mixed effect logistic regression model, with Sub County considered as cluster variable, was used to identify variables associated with presence of mycobacteria in slaughtered pigs. Standard methods were used to assess the fit of the final multivariable model.

## Competing interests

This was purely research work and therefore to the best of our knowledge there was no competing interest.

## Authors’ contributions

AM contributed to the conception, design, and data collection, laboratory work, drafting and writing of the manuscript. CK contributed to laboratory work, data analysis and drafting of the manuscript. TBJ, BD contributed to conception, design, supervision and drafting of the manuscript. VE, DB contributed to the laboratory analysis and drafting of the manuscript. JG and ES contributed to the acquisition of funds, design of study and drafting of the manuscript. All authors have read and approved the final manuscript.
